# Host specialization in ticks and transmission of tick-borne diseases: a review

**DOI:** 10.3389/fcimb.2013.00057

**Published:** 2013-10-04

**Authors:** Karen D. McCoy, Elsa Léger, Muriel Dietrich

**Affiliations:** ^1^MiVEGEC, Mixed Research Unit 5290 CNRS-IRD-UM1-UM2, Centre IRDMontpellier, France; ^2^Department of Biology, Carleton UniversityOttawa, ON, Canada; ^3^Centre de Recherche et de Veille sur les Maladies Emergentes dans l'Océan Indien, GIP CYROISt. Clotilde, France; ^4^Department of Biology, Université de La RéunionSt. Denis, France

**Keywords:** adaptation, Argasidae, *Borrelia burgdorferi* sensu lato, community diversity, epidemiology, Ixodidae, population genetic structure, transmission

## Abstract

Determining patterns of host use, and the frequency at which these patterns change, are of key importance if we are to understand tick population dynamics, the evolution of tick biodiversity, and the circulation and evolution of associated pathogens. The question of whether ticks are typically host specialists or host generalists has been subject to much debate over the last half-century. Indeed, early research proposed that morphological diversity in ticks was linked to host specific adaptations and that most ticks were specialists. Later work disputed this idea and suggested that ticks are largely limited by biogeographic conditions and tend to use all locally available host species. The work presented in this review suggests that the actual answer likely lies somewhere between these two extremes. Although recent observational studies support the view that phylogenetically diverse host species share ticks when found on similar ecological ranges, theory on host range evolution predicts that host specialization should evolve in ticks given their life history characteristics. Contemporary work employing population genetic tools to examine host-associated population structure in several tick systems support this prediction and show that simple species records are not enough to determine whether a parasite is a true host generalist; host specialization does evolve in ticks at local scales, but may not always lead to speciation. Ticks therefore seem to follow a pattern of being global generalists, local specialists. Given this, the notion of host range needs to be modified from an evolutionary perspective, where one simply counts the number of hosts used across the geographic distribution, to a more ecological view, where one considers host use at a local scale, if we are to better understand the circulation of tick-borne pathogens and exposure risks for humans and livestock.

## Introduction

The host range of a parasite, that is, whether it is a host generalist or host specialist, is a vital life history trait that will affect both a parasite's population dynamics and its evolutionary trajectory. Specialist parasites may better track host responses to infection and thus be better able to exploit a host than generalist parasites experiencing diffuse host selection pressures (Whitlock, 1996; Lajeunesse and Forbes, 2002), whereas generalists may be better adapted to cope with environmental variation that affects host community stability (e.g., Kassen, 2002). Host range can also have direct impacts on interacting organisms. For example, generalist parasites can play an important role in host competitive interactions; if one host type suffers more strongly from infection than another, and if those host species compete for resources, infection may tip the balance in favor of the more resistant or tolerant host type (apparent competition; Park, 1948; Holt and Lawton, 1994). When a parasite is also a vector, host range takes on an entirely new dimension because the ability of a vector to exploit one vs. several hosts will not only affect its own population dynamics and evolutionary trajectory, but also that of the associated microparasites.

Ticks are particularly interesting organisms to consider in terms of host range evolution and its consequences. These macroparasites have strong direct effects on host reproductive success and population dynamics, particularly when infestation intensities are high (e.g., Feare, 1976; Duffy, 1983; Boulinier and Danchin, 1996). In tropical zones, they can have significant impacts on livestock production and are a major focus of control efforts (Frisch, 1999; Jonsson, 2006). Ticks also transmit the greatest diversity of pathogenic agents among vector organisms; many of these microparasites are widespread and of considerable medical and veterinary interest (Parola and Raoult, 2001). Understanding the links between host biodiversity, tick host preference and performance, and pathogen transmission are therefore essential for predicting both tick population dynamics and the epidemiology of tick-borne diseases.

Theoretical studies suggest a direct relationship between host diversity and disease risk, but not always in the same direction (Begon, 2008; Johnson and Thieltges, 2010). At a global scale, we expect that increased host biodiversity should lead to increased parasite diversity and exposure (Jones et al., 2008). However, at more local scales, the effect of adding biodiversity can be more variable (Salkeld et al., 2013). With the addition of highly competent hosts for either the pathogen or the vector, or by increasing vector biodiversity, transmission risk may increase (Ogden and Tsao, 2009; Roche et al., 2013). However, the reverse may also be true. In particular, the dilution effect model which predicts reductions in disease risk with increases in host biodiversity, has gained significant popularity over the last decade (Keesing et al., 2006; Randolph and Dobson, 2012). Under this model, different host species within a community vary in their disease reservoir competence; by increasing local biodiversity, competent hosts are diluted among non-competent hosts and the overall density of infected individuals is reduced (Ostfeld and Keesing, 2000). Some empirical evidence for this effect has been found (e.g., Logiudice et al., 2003; Ezenwa et al., 2006; Haas et al., 2011; Johnson et al., 2013), but criticisms to its universality are numerous. For example, it often remains difficult to distinguish between true dilution and an simple reduction in reservoir density when additional species are added to a community (Begon, 2008). Likewise, this model assumes that vector abundance remains the same with increases in host biodiversity (i.e., there is no vector amplification) (Randolph and Dobson, 2012); when vector abundance increases, the number of infected vectors may remain the same, even if infection prevalence is reduced. Finally, and most importantly for this review, the dilution model assumes that vectors are host generalists and exploit both competent and non-competent hosts within a community (Ostfeld and Keesing, 2000). Surprisingly, we have little information on this last aspect, even in well-studied systems. If strong host preferences occur within a community, particularly to the point where vectors have evolved into host races, this model is no longer applicable because, regardless of the diversity of local host species, vectors may tend to use the preferred host. Indeed, host feeding preferences in mosquitoes have been suggested to explain the lack of evidence for the dilution model in some mosquito—West Nile virus systems (e.g., Kilpatrick et al., 2006) and it is becoming increasingly apparent that specific local interactions between reservoir hosts and vectors likely dictate disease risk more than host diversity *per se* (Salkeld et al., 2013).

The aim of the present review is to outline what we currently know about host specialization in ticks and to discuss how this process may affect pathogen circulation. To give proper weight to current ideas, we start with a discussion of the historical notions associated with host range evolution in ticks. We then outline expectations of host use in ticks with respect to previous theoretical work. We review recent studies that have addressed this question using a population genetic approach and show that simple species records are not enough to determine whether or not a parasite is a true host generalist. We then provide a direct example of how host specialization in a tick vector can impact pathogen circulation using Lyme disease bacteria transmission within its marine cycle as a case study. We finish with a discussion of why ticks are exciting organisms to consider in terms of their ability to shift hosts and how the evolution of local host specialization may greatly alter our ability to make reasonable predictions on exposure risk and disease epidemiology.

## Historical views on host specialization in ticks

Many ticks, both hard (Ixodidae) and soft (Argasidae), exploit their hosts for only a short period of time (hours to days) during the bloodmeal and their survival therefore depends strongly on their ability to cope with the conditions of the abiotic environment for the long off-host periods of their life cycle. This dual life style has led to some debate about the relative roles of host and habitat factors in determining both the limits to tick distributional ranges and their evolution.

Based on consideration of specific morphological adaptations, Hoogstraal and colleagues felt that the evolution of tick biodiversity was closely linked to that of their hosts (Hoogstraal and Aeschlimann, 1982; Hoogstraal and Kim, 1985). Hoogstraal and Aeschlimann (1982) considered that at least 700 of the 800 described species of the superfamily Ixodoidea were strict, or relatively strict, host specialists and that this characteristic limited the geographical distribution and population density of most tick species. They suggested that anomalies in recorded data had contributed to an incorrect or ambiguous view of host specificity in this group of parasites. As an example, they note that a tick will secondarily attach to any host type if dislodged during the bloodmeal because its discriminatory senses are dulled or lost and that this behavior may frequently lead to erroneous host records. They do, however, concede that host specificity in ticks is also tightly linked to the ecological characteristics of the host species themselves; hosts that form breeding aggregations have, for example, more specialized ticks, whereas wandering hosts have ticks with either modified life cycles (1 or 2 host ticks) or moderate to low host specificity. These authors further suggested that domestication likely improved conditions for these moderate to low host specific ticks and that their exploitation of livestock has focused our attention on these examples. For them, ticks placed in the “non-particular specificity” category required more intensive investigation, but still remained the exception to the rule.

Klompen et al. (1996) called into question the notion that tick evolution was closely linked to that of their hosts and notably by criticizing the idea that current observations of host-associations in ticks supported host specificity and co-speciation in this group. The authors compiled data from the published literature and found strong positive correlations between the degree of host specificity and sampling effort (number of collections). They felt that this analysis, along with numerous examples of ticks that exploit diverse taxa sharing the same ecological habitat, demonstrated that perceived host specificity in ticks was largely an artifact of incomplete sampling. They suggest that most ticks are not limited by host use, but rather by biogeography; abiotic conditions during the long off-host period of the life cycle. Interestingly, they also remark that recorded host specificity is not a measure of host adaptation *per se*. Although seemingly meant to support the notion that perceived host specialists may not be specifically adapted for the host they are using, the statement can also be interpreted to the contrary that is, perceived host generalists may in fact show specific adaptations to particular host types that are undetected in typical collecting studies.

The view put forth by Klompen and colleagues was supported by a later study that examined ecological ranges of ticks and their hosts. In particular, Cumming (1999) compared the recorded range of different African tick species compiled in a database of published collection records to that of known host species. He found that ~50% of the 229 examined tick species had more restricted ranges than their hosts and that records for the other 50% of species were not complete enough to make any strong conclusions. Only one tick species *Amblyomma rhinocerotis*, a specialist tick of rhinoceros, seemed to conform to the hypothesis that range limits in ticks are determined by their hosts. However, records used in the study did not differentiate between successful and failed host use attempts and did not explicitly consider the relative abundance of ticks on a host or the frequency of records for a given host type. This means that for some species, recorded host ranges may be larger than in reality. Likewise, no consideration was given to local host densities; in some areas of Africa, host densities may be too low to support viable tick populations.

More recently, Nava and Guglielmone (2013) published a meta-analysis of host specificity in Neotropical ticks where they explicitly considered uneven host use and the phylogenetic relatedness of recorded host species [notably by the incorporation of Poulin and Mouillot's (2005) specificity index]. Like Klompen et al. (1996), they found a significant correlation between host range and sampling effort. This relationship was less obvious for the specificity index, supporting the use of this index as a more reliable measure of specificity. Based on their results, no Neotropical tick species was limited to a single host species, and most species used an array of hosts belonging to different families or orders. The index tended to show lower host specificity for immature stages compared to adult stages, a result the authors attribute to host-size constraints that may limit the number of available hosts for adult ticks. More generally, this study concludes that host ecological similarities are more important than host phylogeny in shaping host-parasite relationships in ticks and that ticks tend to be host generalists.

Although these meta-surveys all agree on the primary importance of the abiotic habitat in determining host range and the geographic distribution of ticks, they also all share the same potential pitfalls. First, these studies all assume that tick species are correctly identified using standard morphological characters. However, recent work has shown that the notion of a tick species can be complex (e.g., Estrada-Peña et al., 2012) and many ticks remain poorly described (e.g., Dantas-Torres et al., 2012). Soft ticks are notoriously difficult to identify (Estrada-Peña et al., 2012) and call into question many host records. In addition, many recent revisions have been made that incorporate genetic-based identifications (Guglielmone et al., 2010), but such methods were not employed in historical survey data. Even host range studies that make tick identifications based on conserved genetic markers or traditional morphological characters may miss recently evolved divergences or more cryptic phenotypic changes. More detailed analyses at both genetic and morphological levels can reveal such divergence events (see below). Finally, host specialization in these studies is largely considered from a simple quantitative perception (i.e., number of hosts used). A qualitative framework (i.e., differential performance on different hosts) may provide a more realistic picture of natural interactions and may help us better predict host use and disease risk.

## How specialized should we expect ticks to be?

The specialist-generalist dilemma is founded on the notion “a jack of all trades is master of none.” That is, there is an assumed cost of being adapted to a particular host in that it limits fitness on alternative hosts. This cost and the optimal host range for a parasite will depend on several factors related to the intrinsic characteristics of the parasite, those of the host, and the conditions of the local environment. For example, host availability and predictability is thought to be of prime importance (Jaenike, 1990; Combes, 2001); when hosts are found in high abundance and temporally predictable, parasites should specialize to maximize fitness. Host availability will, of course, depend on both local host abundance, a parameter of the host population, and the ability of a parasite to reach the host through passive or active dispersal, an inherent trait of the parasite. The relative intimacy of the interaction may also dictate optimal specialization; when parasites require a suite of specific traits to overcome physical and physiological barriers to host exploitation, we expect that successful exploitation of a large range of hosts will be difficult (i.e., specialization should evolve). The outcome of ecological specialization can also be modified by local interactions that may favor use of one host over another, such as exposure to predators that alters host–parasite encounter rates (Forister et al., 2012). The mechanistic basis for the presumed cost of adaptation was initially considered to take the form of simple genetic trade-offs; a parasite carrying a certain allele to optimally exploit one host, should suffer lower fitness on alternative hosts (Futuyma and Moreno, 1988). However, the natural world appears more complex and host adaptation likely depends on the genetic architecture underlying a series of traits involved in host use and the potential consequences of gains or losses of particular gene functions (Forister et al., 2012). Both theoretical and field-based studies have suggested that the joint evolution of host preference and performance can greatly favor the evolution of host specialization by restricting the homogenizing effects of gene flow (e.g., De Meeûs et al., 1995; Ravigné et al., 2009; Forister et al., 2012). Gene duplications have also been shown to facilitate expanded resource breadth (e.g., Makino and Kawata, 2012) and may play a role in initiating the evolution of host specificity after a host shift. Finally, these different factors will be conditioned by the relative evolutionary potential of the host and the parasite that is, their respective reproductive rates and generation times (Gandon and Michalakis, 2002).

Several features of ticks may favor the evolution of host specialization (Magalhães et al., 2007). First, ticks engage in a deeply intimate exchange when they exploit their host, particularly in the case of hard ticks where bloodmeals last several days. To maintain blood flow, reduce detection and evade vertebrate immune responses, ticks inject a plethora of bioactive molecules into the host with the saliva (Brossard and Wikel, 2004; Francischetti et al., 2009). This is necessary because innate and acquired immunity in vertebrates can strongly limit tick success (Sonenshine, 1993). For example, Anderson et al. (2013) found a negative association between innate immunity (measured as host blood bacterial killing ability) and the abundance of *Amblyomma hebraeum* and *Rhipicephalus evertsi evertsi* in free ranging African buffalo. Similarly, the development of acquired immunity has been shown to vary significantly among different host species of *Ixodes ricinus* (e.g., Randolph, 1994). The complexity of the tick-host interface should, in principal, limit host range and select for specificity. Similarly, ticks generally have low active dispersal rates and must rely on host movements for among population dispersal (Falco and Fish, 1991; Balashov, 2010). They also frequently emit aggregation hormones that can favor site fidelity (Sonenshine, 1991). These traits will both increase encounter rates with the same host type and limit gene flow between populations that may be undergoing diffuse selection pressures. At the extreme, certain tick species may remain on a single host individual for the entire life cycle (one-host tick), dropping off the host only to lay eggs (e.g., *Rhipicephalus microplus*); in such cases, one may expect strong selection for host specialization (see below). Reproductive potential should also be greater in ticks than in their hosts; ticks can lay anywhere between 500 and 20,000 eggs after repletion, with hard ticks laying all eggs in a single bout and soft ticks spreading laying out over several short bloodmeals (Sonenshine, 1991). Generation times likewise vary between 1 and 6 years (Sonenshine, 1993), and although long for a parasite, they will frequently be shorter than for the associated vertebrate hosts. The last factor, related to the underlying genetic architecture of adaptation in ticks, is more complicated and we are only in the very initial stages of understanding genetic evolution and adaptation in ticks. In general, tick genomes are large (one-third to two times the human genome) with a high percentage of repeated elements (Nene, 2009; Meyer et al., 2010). The presence of high quantities of mobile elements may, on the one hand, generate high background genetic variation on which selection for specialization may act, but they may also break apart adaptive gene complexes that favor adaptation to specific hosts (e.g., Sunter et al., 2008; Casacuberta and Gonzalez, 2013). Results of genome sequencing projects on focal tick species should improve our understanding of how genetic architecture modulates the evolution of host specialization in ticks (Hill and Wikel, 2005; Guerrero et al., 2006).

## Integration of population genetics to study host use

When considering the evolutionary ecology of ticks and their associated pathogens, the notion of host range has to be modified from an evolutionary perspective, where one considers counts of the number of hosts used across the geographic distribution, to a more ecological perspective where one considers host use at a local scale and its implications in terms of co-evolutionary interactions and pathogen circulation. Indeed, field observations suggest that even broad host generalists tend to feed on only a few main hosts locally, with these hosts changing across different areas of the distribution (e.g., Balashov, 2010). This type of local thinking can provide key information on the relative ease of host switching in these species and how this may modify disease risk. With the development of genetic and genomic tools, we can now have more ready access to information on host use and specialization in vector-borne disease systems at local scales, going beyond simple records of observed host use. In particular, population genetic approaches that employ neutral genetic markers, in combination with information on host use, can enable us to determine whether ticks have locally diverged into reproductively isolated units that exploit specific host types (McCoy, 2008). Even host use information can be obtained indirectly via modern molecular methods (e.g., amplification and identification of bloodmeal traces; Kent, 2009) in cases where direct host sampling is not possible. This information can then be related to pathogen prevalence estimates in both hosts and vectors to infer transmission pathways and disease risk. Only a few such studies on ticks have been conducted to date, but all have revealed significant patterns of local host-associated genetic structure in ticks. We briefly review these examples in the following paragraphs.

**Figure 1:**
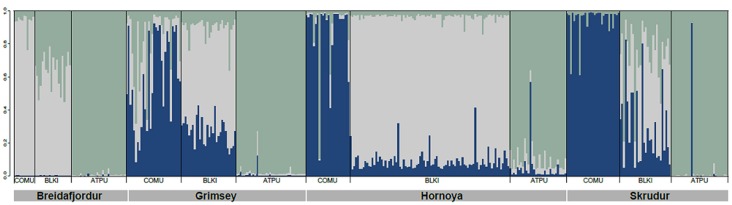
**Host-associated genetic structure in the seabird tick *Ixodes uriae* from four North Atlantic mixed colonies.** The data for 8 microsatellite markers are re-analysed from Kempf et al. (2009a). The number of genetically distinct pools of individuals present within the sampled populations was determined using the Bayesian clustering approach implemented in STRUCTURE v.2.3.1 (Pritchard et al., 2000). The number of potential clusters (K) was set from 1 to 13, with 5 independent runs. Computations were run under the admixture model with correlated allelic frequencies and sampling location as a prior. Simulations were carried out using a burn-in of 100,000 iterations, followed by a run length of 100,000 iterations. The most probable number of populations was 3. Each tick individual is represented by a thin vertical bar composed of K segments whose length is proportional to the probability that the tick belongs to each genetic pool (here, respectively blue, light gray, and green for the 3 groups). The seabird hosts present in each colony are shown below the colony name and are abbreviated as in Table 1. In all colonies, there is significant genetic structure among the 3 tick groups (Kempf et al., 2009a) with the appearance of a well-defined genetic race for ATPU ticks. For COMU ticks, the Breidafjordur colony showed a different pattern of group membership than the other 3 colonies, with COMU ticks being more closely related to BLKT ticks of the other colonies. In this colony, the BLKT ticks are an admixture of the other two races.

The cosmopolitan seabird tick *Ixodes uriae* was the first tick system studied to test for the presence of host-associated population genetic structure. This hard tick exploits nesting colonial seabirds in the circumpolar regions of both hemispheres and was considered to be a seabird generalist, with more than 60 different host species recorded (Dietrich et al., 2011). *I. uriae* is a nidicolous (i.e., nest-inhabiting) tick and is only associated with its seabird host for the bloodmeal which it takes once in each of its three life stages (larva—nymph—adult; adult males do not feed) for a period of between 3 and 12 days, the length increasing with successive life stages (Eveleigh and Threlfall, 1974; Frenot et al., 2001). The remainder of its life is spent in the substrate surrounding the host nesting area, often in aggregates of several hundred individuals (Benoit et al., 2007). This tick reproduces sexually and a female will lay several hundred eggs in a single bout before dying (Eveleigh and Threlfall, 1974). A diverse array of viruses and bacteria circulate in seabirds via *I. uriae*. Some are clearly pathogenic for humans, like the bacteria of the *Borrelia burgdorferi* sensu lato (Bbsl) complex responsible for Lyme disease (see below), whereas others are largely limited to seabirds with unknown effects for these hosts or for humans (Dietrich et al., 2011). After observing asynchrony in the timing of tick exploitation on different sympatric seabird species, McCoy et al. (1999) hypothesized that *I. uriae* populations may consist of a series of local host-specific groups. They tested the predictions of this hypothesis using a population genetic approach with specifically-developed microsatellite markers (McCoy and Tirard, 2000) and found that throughout the different zones of its global distribution, this tick had indeed formed host-specific genetic groups (or host races) and that these host races had evolved independently in different isolated regions (McCoy et al., 2001, 2005, 2012; Dietrich et al., 2012). This divergence has been suggested to be relatively recent (Kempf et al., 2009a), but has been accompanied by both phenotypic changes in body morphology (Dietrich et al., 2013) and host-associated variation in performance on different host species (Dietrich, 2011). An example of local patterns of host-associated divergence in this tick is shown in Figure 1. For this analysis, ticks were sampled from three different seabird hosts breeding within four large colonies and were genotyped at a series of microsatellite markers. All tick individuals were included in a global clustering analyses (see figure legend for details) and the main pattern found shows that ticks using different local seabird host species tend to be genetically distinct; there are clear patterns of within-colony structure among host-associated tick groups, with some evidence of occasional admixture between groups and large scale dispersal among locations.

*Rhipicephalus* (*Boophilus*) *microplus* is a widely distributed, one-host tick that exploits livestock (mainly *Bos indicus, B. taurus*, and *Equus caballus*) and wild ruminants in subtropical and tropical regions. This tick originated in Asia, but during the second half of the 19th century spread via cattle transportation to Australia, Madagascar, South Africa, Latin America, Mexico, and the USA (Cumming, 1999). Since this time, it has continued to spread across the different invaded continents (e.g., detected in West Africa in 2007; Madder et al., 2007). This tick causes significant damage to livestock production across its distribution (Frisch, 1999), including the transmission of numerous diseases of medical and veterinary importance such as bovine and equine babesiosis (Apicomplexa: *Babesia bigemina, B. bovis, B. caballi*) and anaplasmosis (Proteobacteria: *Anaplasma marginale*). However, the degree to which *R. microplus* uses native wildlife and the potential reservoir status of these alternative host species is unclear. Chevillon and colleagues recently tracked the invasion of this tick in New Caledonia (Koffi et al., 2006) and showed that despite a strong initial bottleneck and, after only ~240 generations since its arrival on the island, the tick had diverged into two well-defined host-specific groups with little to no genetic exchange, a race that exploits cattle and another that exploits the rusa deer (*Rusa timorensis*) (De Meeûs et al., 2010). However, the general tendency for host-associated divergence across the vast distribution of this tick is still unknown. Indeed, in New Caledonia, acaricide use is extremely high and may have favored the rapid divergence of host-associated groups (Chevillon et al., 2007). In other areas of the world, acaricide use may be more or less restricted, but local host-related structure has never been tested. What is clear from this initial study is that, despite its recent colonization history and the continued presence of its ancestral host species in newly invaded zones, *R. microplus* can rapidly evolve host specificity to novel host species and this specificity may greatly alter its population dynamics and the transmission of pathogens between livestock and wildlife.

**Figure 2:**
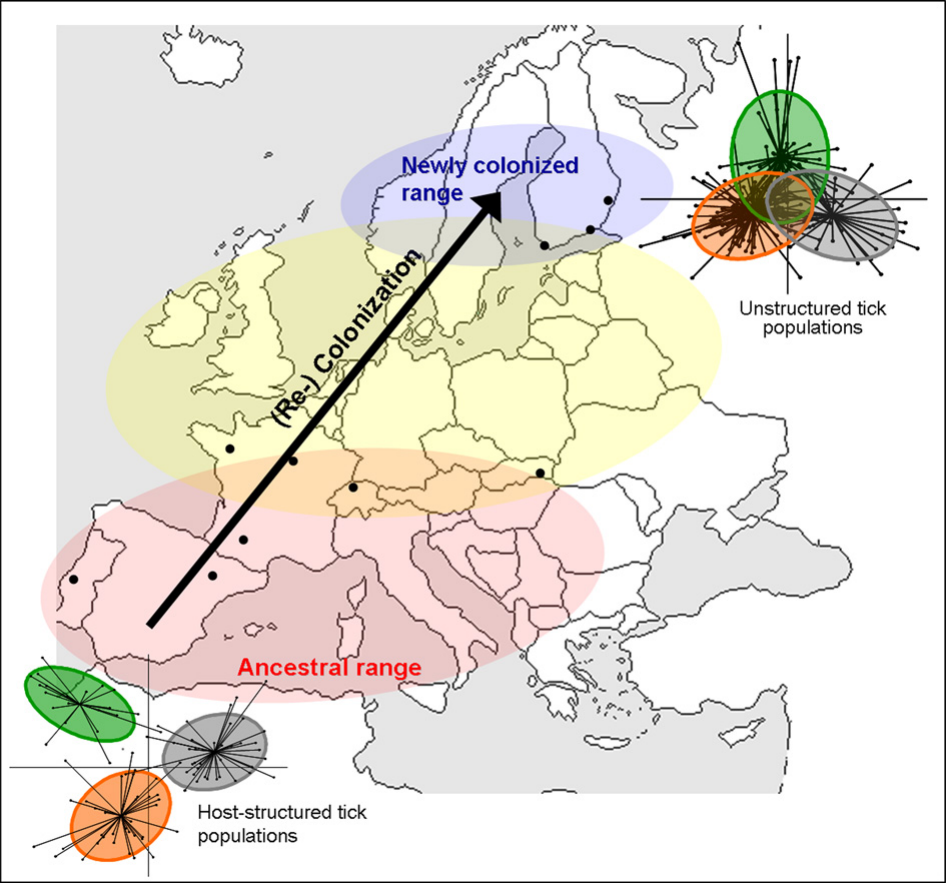
**Schematic representation of population expansion of *Ixodes ricinus* (arrow) and hypothesized consequences for host-associated genetic structure.** The red zone represents well-established *I. ricinus* populations that persisted in both Southern and Central Europe during the last glacial phase (Porretta et al., 2013). The blue area represents the recently colonized zone for *I. ricinus* and the yellow zone, a transition area where population age and history may be variable (Léger et al., 2013; Porretta et al., 2013). Under the hypothesis that the evolutionary age of a tick population may affect the evolution of host specialization, we would expect a strong pattern of specialization in the red zone, and no host specialization in the blue zone because ticks have only been exposed to local hosts for a few generations. In the yellow zone, patterns of host specialization may be more variable. On the figure, the degree of host-associated population structure of *I. ricinus* in the extreme zones are represented by a between-group analysis of neutral genetic variation, where each dot represents an individual tick and the color indicates different host-associated tick populations. Greater separation of groups indicates stronger genetic divergence. The relative evolutionary age of tick populations could be changed to some other habitat-based factor that may affect the evolution of host specialization.

The third example comes from *Ixodes ricinus*, the principal European vector of Lyme disease and other major pathogens of human interest (*Babesia* spp, Tick-borne encephalitis virus, *Anaplasma phagocytophilum*, etc.). This tick has a wide distribution across western Europe and, due to global change, is currently expanding its range northward and to higher altitudes (see review of Léger et al., 2013). *I. ricinus* is found in deciduous woodlands and mixed forests, where it is highly sensitive to temperature and humidity, and where it is typically active from spring to autumn (Gray, 1998). The general life cycle is similar to *I. uriae*, but this tick actively quests on the vegetation for its host. It is commonly recognized that host specificity of the different life stages of *I. ricinus*, and related tick species, is linked to host size constraints; larvae and nymphs parasitize almost all vertebrate size classes, whereas female adults only feed on larger mammals (e.g., Eisen and Lane, 2002). Indeed, this hard tick is touted as the example *par excellence* of a host generalist, parasitizing a vast range of terrestrial vertebrates including mammals, birds, and reptiles (Sonenshine, 1991, 1993). Recent work on the population genetics of *I. ricinus* nonetheless suggests that some host specificity may evolve within local communities, other than that related to size constraints. Previous studies have indicated a lack of population genetic structure at large spatial scales (Delaye et al., 1997; De Meeûs et al., 2002; Casati et al., 2008; Noureddine et al., 2011), but strong patterns of heterozygote deficits within populations, a potential indicator of local substructure (De Meeûs et al., 2002; Kempf et al., 2010). Indeed, a reanalysis of data from De Meeûs et al. (2002) that took local substructure into account showed significant patterns of isolation by distance among Swiss populations of *I. ricinus* (De Meeûs, 2012). Likewise, patterns of mate choice suggest the presence of assortative mate pairing in some populations (Kempf et al., 2009b). Kempf et al. (2011) measured genetic variation at microsatellite markers in a large sample of ticks collected directly from sympatric host types (birds, rodents, lizards, wild boars, and deer) in several European locations and observed significant genetic structure among ticks from different host types, but only in certain populations. This suggests that host choice is not random for *I. ricinus* and that host preferences may evolve in local populations and be linked to mate choice. However, as different areas of the distribution seem to show different degrees of host-related divergence and different tick life stages have host-associated feeding constraints, these observations require further investigation in order to fully understand the specialization process in this species. The evolution of host associated divergence may vary, for example, with the history and composition of local host communities (Kempf et al., 2011). Under this hypothesis, we could predict that longer established and/or more stable host communities should show stronger patterns of host-associated divergence than more recently colonized or perturbed host communities (Figure 2). The formation of specialized host races in *I. ricinus* would not only profoundly alter our understanding of how populations of this tick function under natural conditions, but would also represent a significant transmission constraint for the different pathogens it vectors. The existence of such patterns would thus require explicit consideration in epidemiological models of tick-borne disease.

Finally, an initial population genetic study of a group of soft ticks that exploit marine birds, ticks of the *Ornithodoros capensis* complex, has also suggested that local host specialization may evolve in ticks (Gómez-Díaz et al., 2012). As mentioned above, soft ticks have fundamentally different life cycles than hard ticks and may be less intimately associated with the host due to their numerous, but brief, encounters for the bloodmeal. The *O. capensis* system is interesting in that it parallels the *Ixodes uriae* system in terms of host characteristics, but has a complementary geographic distribution, covering temperate to tropical regions of both hemispheres, rather than polar zones (Dietrich et al., 2011). A detailed study based on ticks collected from different seabird species breeding sympatrically in colonies of the Cape Verde Archipelago and typed at a conserved mitochondrial genetic marker (16S) and a single nuclear marker (18S) showed initial indications of host-associated divergence. Indeed, five sympatric lineages of ticks were found within the island archipelago, many well-outside their described geographic distribution, suggesting wide-scale dispersal of these ectoparasites. However, a detailed analysis of haplotype structure within a single lineage revealed patterns of divergence among ticks exploiting different sympatrically breeding seabird host species (Gómez-Díaz et al., 2012).

Interestingly, observational and experimental results in North American ticks may also support the presence of local tick specialization. For example, the relative infestation prevalence of *Ixodes scapularis*, the main vector of Lyme disease in the eastern US, on rodent and lizard hosts shifts from north (on rodents) to south (on lizards), potentially in relation to relative host abundance (e.g., Apperson et al., 1993; Durden et al., 2002). In the western US, Swei et al. (2011) carried out a removal experiment where the main host of *Ixodes pacificus*, the western fence lizard (*Sceloporus occidentalis*), became unavailable to questing larvae. Rather than immediately switch to alternative hosts, a higher than average number of questing larvae was maintained in the population over the course of the season. There was likewise no obvious increase in larval infestation rates on surveyed deer mice and only about a 5% increase on female wood rats. This could suggest that larvae continued questing for lizard hosts after their removal. In the following year, the authors observed a significantly lower density of questing nymphs, supporting the hypothesis that questing larvae never found appropriate hosts when lizards were unavailable. Population genetic studies of both of these systems could be particularly revealing to understand whether observed host use represents a highly plastic behavior, or whether genetic divergence has occurred among ticks that exploit different local hosts. Indeed, an initial genetic study in *Dermacentor albipictus*, a one-host tick of North American ungulates, suggests the evolution of host specialization among different sympatric host species, despite the large dispersal potential of both wild and domestic hosts (Leo, 2012).

## Consequences for pathogen circulation

**Figure 3:**
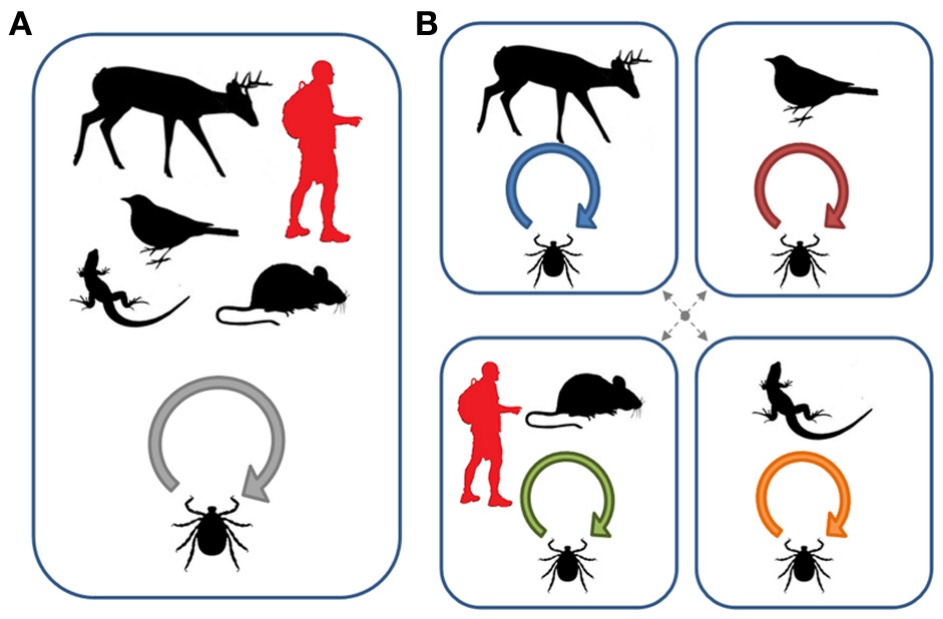
**Pathogen circulation within host communities depending on vector specialization. (A)** Several host species are available locally, but all are equally used by the vector (i.e., the vector is a true generalist). The local transmission and evolution of the microparasite will therefore depend on the abundance of each host species and the relative selection pressures they impose. Under this scenario, humans are exposed to the general vector population and surveys of disease prevalence in this general population will be a reasonable measure of risk. **(B)** Each vertebrate host species is exploited by a distinct vector population resulting in the presence of locally independent or semi-independent disease cycles. Dotted arrows refer to incomplete host-associated isolation. The evolutionary outcome of these interactions will depend on the degree of specificity of each vector population and the ability of the microparasite to exploit alternative hosts after transmission. Human exposure in this type of system will also depend on vector specificity and the cues used for host selection (in the diagram, humans are primarily exposed to ticks that exploit small mammals). Under these conditions, a survey of infection prevalence from the overall tick population may significantly under- or over-estimate exposure risk (Figure after McCoy, 2008).

Many pathogens that cause human or livestock disease are maintained in natural foci which then spillover to humans or their domesticated animals (Balashov, 2010). As vertebrate species may vary greatly in their reservoir competence for different pathogens, host specificity of vectors becomes an essential issue for developing predictive models of disease risk (such as the dilution effect model mentioned above). As the host specificity of vectors may be more of a local scale phenomenon, an ecological view of this aspect is the most appropriate to incorporate into studies of pathogen circulation. Figure 3 illustrates how changes in local host use can alter patterns of pathogen transmission and exposure risks for humans. In particular, if ticks form naturally-occurring host-associated groups, human exposure risk will depend both on the degree of vector specificity for each host type and on the cues that different tick groups use in order to select their particular host. Humans, as incidental hosts, may only be exposed to host groups that share similar cues for questing ticks (such as mice in Figure 3). Under these conditions, estimates of infection prevalence derived from the overall tick population, collected by flagging for example, may significantly under- or over-estimate exposure risk.

An illustration of the consequences of vector specialization on pathogen circulation can be found if we return to the *Ixodes uriae* system and examine patterns of prevalence and diversity in Lyme disease bacteria (*Borrelia burgdorferi* sensu lato—Bbsl) circulating among different host-associated tick populations within seabird colonies. Detailed analyses of tick infections by Bbsl indicated significant differences in local prevalence estimates among different sympatric tick populations (Gómez-Díaz et al., 2010; Table 1). While little variation exists among host groups in terms of the presence of different Bbsl genospecies (Table 1), significant genetic structure is evident if one looks at strain variation within a given genospecies (Table 2). *B. garinii* is the dominant genospecies found in the marine system and, although geographic structure is low among circulating strains (Gómez-Díaz et al., 2011), host-associated structure is high within most colonies (Table 2) and demonstrates that the evolution of host specificity can be a major barrier to local transmission. Results from the *Ixodes uriae* system may have limited direct effects for Lyme disease epidemiology *per se* (although some *B. garinii* strains are exchanged with terrestrial systems; Gómez-Díaz et al., 2011), but similar patterns occurring in terrestrial vector-borne disease systems may call into question our current thinking on the transmission ecology of specific pathogens. For example, in terrestrial systems, different genospecies of the Bbsl complex are associated with different host types, a pattern that is thought to be maintained by host complement responses to the bacteria (Kurtenbach et al., 2002, 2006). However, as pathogen transmission depends both on the ability of the host to carry the infection and on host use and infectivity of the vector, it is possible that vector specialization may have favored the evolution of these associations, or at least, may play a role in their maintenance. Recent theory also predicts a significant role of vector diversity in driving epidemiological patterns of associated disease. Roche et al. (2013) used a theoretical reservoir-vector-pathogen framework to study the transmission consequences of increasing host reservoir and/or vector species richness within the context of large community assemblages. They found that increasing vector diversity, regardless of the variance in infectivity among these vectors, could increase overall disease transmission in the system because increases in vector richness tend to lead to a greater overall abundance of potential vectors. However, this tendancy is modified if vectors specialise on different host types. Clearly, it appears that if we are to better understand the evolution and epidemiology of vector-borne diseases, we need to explicitly test whether perceived generalist vectors are true generalists or rather composed of a diverse assemblage of cryptic host specialists.

**Table 1:** **Borrelia prevalence and distribution among *Ixodes uriae* ticks from different seabird species breeding in four seabird colonies of the North Atlantic**.

**Colony (coordinates)**	**Host species (nb sampled)**	**Nb. Ticks (Ad, Ny)**	% Prev^*^	Borrelia species^**^				
				**Bg**	**Bl**	**Bbss**	**Ba**	**Co**
Hornøya, Norway (70°22′N, 31°10′E)	BLKI (259)	380 (265, 115)	10.3	22	0	7	1	0
	ATPU (62)	92 (67, 25)	31.8	17	0	5	3	0
	COMU (52)	107 (105, 2)	17.8	14	0	1	1	0
Skrudur, Iceland (64°54′N,13°38′W)	BLKI (17)	28 (25, 3)	17.9	2	2	0	0	1
	ATPU (19)	31 (26, 5)	22.6	7	0	0	0	0
	COMU (27)	32 (23, 0)	18.8	4	2	0	0	0
Grimsey, Iceland (66°33′N, 18°00′W)	BLKI (24)	30 (16, 14)	33.3	5	2	0	0	3
	ATPU (28)	39 (29, 10)	33.3	11	2	0	0	0
	COMU (23)	30 (25, 5)	33.3	5	3	0	0	2
Breidafjordur, Iceland (65°23′N, 22°54′W)	BLKI (11)	20 (2, 18)	45.0	6	2	0	0	1
	ATPU (25)	30 (14, 16)	63.3	13	0	0	0	2
	COMU (7)	12 (12, 0)	25.0	1	0	0	0	1

Host species include Black-legged kittiwakes Rissa tridactyla (BLKI), Atlantic puffins Fratercula arctica (ATPU), and Common murres Uria aalge (COMU). Bacteria of the Borrelia burgdorferi sensu lato complex in I. uriae were characterized using a nested PCR procedure followed by direct sequencing of a FlaB gene fragment. The species found include B. garinii (Bg), B. lusitaniae (Bl), B. burgdorferi sensu stricto (Bbss), and B. afzelii (Ba). The species could not be clearly identified in the case of co-infections (Co). See Duneau et al. (2008) for details on the molecular analysis.

* May be slightly overestimated because some ticks are from the same host individual.

** Not all positive amplifications could be successfully sequenced for species identification.

**Table 2:** **Analysis of Molecular Variance (AMOVA) results for *B. garinii* isolated from *Ixodes uriae* ticks sampled from three different seabird host species in four mixed species breeding colonies in the North Atlantic (see Table 1 for details)**.

**Component**	***df***	**% Variation**	**Φ-statistic**	***P*-value**
Among colonies	3	6.31	0.06307	0.16227
Among host races within colonies	8	14.01	0.14955	**<0.001**
Within host races	94	79.68	0.20319	**<0.001**

The analysis specifically tests for genetic structure among isolates both among tick host races within colonies and among colonies. The Φ-statistic provides an estimate of the genetic structure among isolates at each hierarchical scale and takes into account both the molecular distance among sequences and their frequencies (Excoffier and Lischer, 2010). P-values are based on permutation procedures specific to each hierarchical level and indicate the significance of each component (significant values in bold). Analyses are based on a 308 bp fragment of the FlaB gene (see Duneau et al., 2008, for details). An alternative grouping with colonies nested within tick races provided similar results.

## Conclusions and perspectives

Our goal in this review was to clarify our current thinking on the evolution of host specialization in ticks and to consider how this process may alter patterns of tick-borne disease transmission. Historical notions of host specialization in ticks have been contradictory in terms of whether ticks tend to be host specialists or host generalists. However, by re-evaluating the spatial and temporal scales considered in analyses of host use, both views can be supported.

At the scale of the global geographic distribution of a species, ticks tend to be host generalists. Most species have large repertoires of potential host species and can exploit phylogenetically diverse host species that share the same ecological habitats. Studies in other ectoparasites likewise suggest that the ecological similarity of the host environment may be more important than host phylogenetic similarities in determining a parasite's host range (Krasnov et al., 2010). Ecological fitting, the ability of an organism to colonize and form novel associations, therefore seems to be an appropriate framework for understanding tick host use at large spatial scales (Agosta and Klemens, 2008). However, given the complexity of the tick-host interface and the life history characteristics of these ectoparasites, the ability to exploit a highly diversified group of hosts seems counter-intuitive. How can one account for the high plasticity in host use that seems to be maintained by ticks? The genetic architecture of tick genomes may provide some clues to this apparent flexibility; its relatively large size and repetitive nature may provide ticks with the diversity and/or redundancy required to rapidly exploit novel hosts (Sunter et al., 2008; Nene, 2009). Detailed analyses from current tick genome projects should provide some data to examine this question more directly (Hill and Wikel, 2005; Guerrero et al., 2006).

At a more local scale, host specialization seems to be the norm in ticks, at least in the tick systems studied in detail so far. These species are able to rapidly form distinct host-associated populations within local communities and these different populations can show differences in both host preference and performance. In this sense, simple records of host species observations are not sufficient to determine whether a tick species is a specialist or a generalist; detailed information on patterns of host use is required. The local scale is also the appropriate one to consider if we want to better predict pathogen circulation and exposure risk to humans and their domestic animals, because it is at these spatial scales that transmission takes place. In order to explicitly take into account this contrast in host use between local and global scales, a more suitable measure of host specialization in tick species would be to estimate turnover (or beta diversity) in host use across the geographic range (Krasnov et al., 2011).

The question remains as to the fate of local specialist, global generalist ticks. Do ticks which diverge into local host-associated genetic groups continue toward speciation or is perceived adaptation based simply on phenotypic plasticity and ecological fitting? The evolution of specialization is considered to be an essential step toward speciation (e.g., Maynard Smith, 1966; Schluter, 2000). Analyses in phytophagous insects have often shown positive correlations between the number of distinct species within taxa and the relative degree of plant specialization (e.g., Dyer et al., 2007; Forister et al., 2012). If ticks frequently evolve host specialization and this leads to speciation, we should also expect higher species richness in those genera that tend to show more stringent host preferences, even if host shifts frequently occur. We should also expect that sympatric host races should become increasingly isolated over evolutionary time to the point of becoming distinct entities (e.g., evolve sustainable adaptive diversity; De Meeûs et al., 2003). Current data on these aspects is greatly lacking. A partial analysis of the African tick database compiled by Cumming (1998) suggests a positive correlation may exist between the number of tick species within a given genera and their recorded number of hosts (e.g., for mammalian host types; *r* = 0.78, *P* < 0.001). However, in the *Ixodes uriae* system, seabird host races are not more divergent in the ancestral range of the species compared to more recently colonized regions (McCoy et al., 2005; Dietrich et al., 2012) suggesting that the race state may be maintained over long time periods. Field-based studies on a larger range of tick species will be required to examine these hypotheses in more detail. Genetic studies that examine host and vector co-structures would also reveal the potential role of on-going gene flow in the long-term maintenance of host-associated genetic races.

The question of host specialization in ticks is an important one for understanding their evolution, their population dynamics and the circulation and diversification of the parasites that they transmit. Ticks can be particularly challenging parasites to work with because they have relatively long life cycles, are often difficult to maintain under laboratory conditions and have complicated genetic architectures. However, their complex life histories, remarkable adaptations for host exploitation, and importance for the transmission of a diverse range of pathogens renders them exciting models for addressing such questions. With increasing advances in genetic technologies, data to address many of questions raised in this review should become available. With a better understanding of the evolution of host specialization over ecological and evolutionary time, the planning of effective tick control programs and our understanding of tick-borne disease circulation and emergence should greatly improve.

### Conflict of interest statement

The authors declare that the research was conducted in the absence of any commercial or financial relationships that could be construed as a potential conflict of interest.
